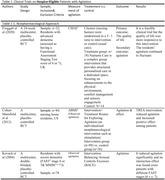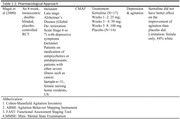# A Literature Review of Randomized Controlled Trials for Hospice‐eligible Patients Living with Dementia and Agitation

**DOI:** 10.1002/alz.095077

**Published:** 2025-01-09

**Authors:** YuHsuan (Olivia) Wang, Arianne Fritts, Doris P. Molina‐Henry, Joshua D Grill, Jacobo Mintzer, Rema Raman

**Affiliations:** ^1^ USC, Los Angeles, CA USA; ^2^ Medical University of South Carolina, Charleston, SC USA; ^3^ Alzheimer’s Therapeutic Research Institute, University of Southern California, San Diego, CA USA; ^4^ Institute for Memory Impairments and Neurological Disorders, University of California, Irvine, Irvine, CA USA

## Abstract

**Background:**

Most hospice‐eligible patients with dementia report agitation with antipsychotics and antidepressants with major side effects predominantly used in this population to control symptoms. Little is known about current evidence on randomized controlled trials (RCTs) on reducing agitation in this population.

**Objectives:**

We aimed to summarize and evaluate the existing literature on RCTs aimed at reducing agitation among hospice‐eligible patients with dementia.

**Methods:**

We conducted a literature review using the following keywords (end‐of‐life, late‐stage dementia, agitation, terminal restless, terminal delirium, randomized controlled trials) on MEDLINE, Pubmed, PyscINFO, Proquest, CINAHL, EBSCO, EMBASE, Cochrane Library, and references of previous publications. This review considered English language RCTs and review articles on RCTs that focus on interventions for reducing agitation among patients who were terminally ill, reported symptoms of agitation, and were diagnosed with dementia.

**Results:**

The search included published articles from January 1990 to February 2024. Forty‐four articles were identified. Of these articles, sixteen RCTs did not include terminally ill patients with dementia and agitation; seventeen did not focus on reducing agitation; four were not completed trials, two were written in German, and one could not be retrieved. Of the four relevant articles included in this review, three tested a nonpharmacological intervention and one tested a pharmacological intervention. The sample sizes in all four studies were small: ranging from 30 to 90 participants. Of the three studies that reported race, more than 75% of participants were White. None of the studies reported Latino ethnicity. Only one study could apply to the last five‐year period, but the use of agitation reduction was the secondary outcome of the study. No interventional trials with a pharmacological approach have been reported in the previous twenty years.

**Conclusion:**

Our literature review identified only four RCTs conducted in hospice‐eligible patients with agitation in the last four decades with no medication being evaluated in over a decade. Research is urgently needed to identify, develop, and test treatments for end‐of‐life agitation in patients with dementia.